# CSI: Bethesda

**DOI:** 10.1371/journal.pbio.0040296

**Published:** 2006-09-12

**Authors:** Philip Shaw

## Abstract

Philip Shaw reviews *Visible Proofs: Forensic Views of the Body,* the current exhibition at the National Library of Medicine in Bethesda, Maryland.

**Figure:**
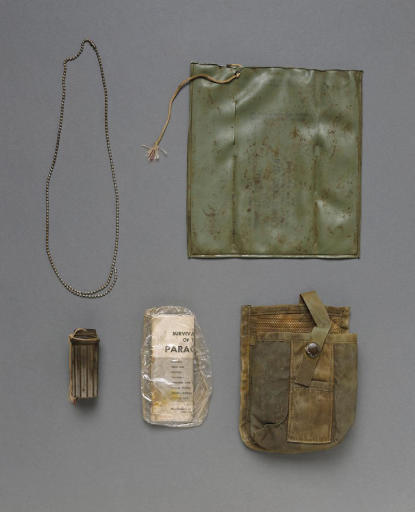
Air Force gear found with former unknown soldier Michael Blassie, from “Visible Proofs,” on exhibition at the National Library of Medicine through 2008 (clockwise from top left: dog tag chain, signal marker pouch, match holder, parachute survival guide, and ammunition pouch) (courtesy of the National Library of Medicine)

The most popular television programs in the US, such as *CSI*, are based on forensic medicine, the science of investigating violent or suspicious deaths to gather evidence for criminal or civil cases. “Visible Proofs: Forensic Views of the Body,” the current exhibition at the National Library of Medicine in Bethesda, Maryland, is thus timely and in keeping with the institution's mission of demonstrating how science informs and reflects contemporary interests.

Forensic medicine arose in the 16th century as a hybrid outgrowth of the increasingly organized professions of medicine and law, and it was fueled by Renaissance scholars' fascination with the human body. Though precursors of the discipline are found in ancient Chinese texts and in a few suggestive Egyptian hieroglyphs, the English get the credit for creating the post of coroner, who was required to make a “view of the body,” a formal visible inspection in cases of murder or suspicious deaths.

“Visible Proofs” traces the evolving technologies applied to “seeing into the body”—making evidence from the traumatized body visible in a way that can implicate the guilty and exonerate the innocent. The exhibition starts with unaided visual inspection of surface anatomy: looking at the signs of strangulation, entry wounds, and patterns of decomposition. It moves to postmortem dissection, with displays of the anatomist's tools, including those used in President Abraham Lincoln's autopsy. The central display is an autopsy teaching video for forensic pathologists that starts with a gutting Y incision across the chest and abdomen and then proceeds to evisceration. While the video may delight the ghoulish schoolchild in us all, its clinical precision and accompanying monotone commentary tempt the viewer to forget that the subject being dissected was a human being—a man who died of a drug overdose and whose identity was known. An interactive virtual autopsy, found just nearby, allows you to direct which bits of the body you want to “dissect” and is perhaps better suited to the faint-hearted.

As you move further into the displays, the level of analysis deepens, passing first to the staple of Victorian novels: toxicological techniques used primarily to identify poisons. The evolution of tests used to detect poisoning by arsenic and heavy metals is much less interesting than the press descriptions of court appearances by testifying chemists, who apparently had a highly developed sense of dramatic timing and theatricality. By the late 1800s, other biophysical methods such as spectroscopy were being applied to match blood and other elements found on victims with their assailants. These techniques remain important: in the relatively recent prosecution of the respiratory therapist Efren Saldivar, the exhumation and testing of bodily remains for pancuronium bromide (a paralytic agent that was not prescribed for these patients by their treating physicians) provided strong evidence that Saldivar had deliberately administered this potentially harmful drug to patients.

Molecular science techniques are given deserved weight, and the strengths of these technologies are clear. Kirk Bloodsworth, who opened the exhibition, was wrongly imprisoned for the sexual assault and murder of Dawn Hamilton, a 9-year-old girl. After a long battle, his lawyer persuaded the police to allow comparison of DNA from his blood with DNA found in sperm on the body. The DNA evidence cleared Bloodsworth, who now remains active in projects that advocate the reform of the American judicial system to prevent systematic miscarriages of justice. DNA matching also allowed the identification of the unknown soldier from the Vietnam War as Lt. Michael Blassie.

The exhibition is resolutely positive about the contribution of forensic science to the judicial process. This extends to the arena of human rights: there is a display detailing the efforts of the application of its techniques to the excavation of clandestine mass graves in Argentina, a process that has been pivotal in bringing several members of Argentina's military regime to justice for human rights violations.

There are other delights: maggots found on decomposing bodies and dollhouse reconstructions of real murder scenes (which are still used for teaching medical examiners). The pamphlet *A True Relation of a Barbarous Bloody Murther*, published in 1688, details how investigators in the case used a forensic test based on the ancient belief that the corpse of a victim will bleed if touched by the murderer, a salutary reminder that not all forensic science has been good science.

If a trip to Bethesda is not for you, there is a beautifully constructed Web site that contains most of the core elements of the exhibit itself (http://www.nlm.nih.gov/visibleproofs).It allows you to navigate between key cases in the development of forensic science, the main technologies involved, and biographies of the leading lights in the field. The media section is the most interesting and includes teaching videos of two autopsies, which take the viewer from the initial incisions through to the dissection and analysis of individual organs. There is also slightly surreal footage from the Ragsdale gunshot wound study from the 1980s, which shows the effects of a bullet from a Colt revolver being shot into a human bone set in Jell-O. The rather more devastating effects of a M-16 submachine gun can only be seen in the exhibition itself.

It's hard to explain the proliferation of *CSI*-style programs and why this exhibition seems so apt. It may reflect a response to a society in which medicine and the funerary industry have “professionalized” the process of dying, making it a more remote experience. As the guide to the exhibit hints, the displays may thus not merely inform a fascination with the application of science to crime, but feed a deeper interest in the reality of death.

